# Daptomycin for the treatment of osteomyelitis and orthopaedic device infections: real-world clinical experience from a European registry

**DOI:** 10.1007/s10096-015-2515-6

**Published:** 2015-11-13

**Authors:** K. Malizos, J. Sarma, R. A. Seaton, M. Militz, F. Menichetti, G. Riccio, J. Gaudias, U. Trostmann, R. Pathan, K. Hamed

**Affiliations:** Orthopaedic Department, School of Medicine, University of Thessalia, Larissa, Greece; North Tyneside General Hospital, North Shields, UK; Queen Elizabeth University Hospital, Glasgow, UK; Department of Septic and Reconstructive Surgery, Trauma Center Murnau, Murnau, Germany; Azienda Ospedaliera Universitaria Pisana, Ospedale Cisanello, UO Malattie Infettive, Pisa, Italy; Infectious Diseases Hospital Santa Maria della Misericordia Albenga ASL-2, Albenga, Italy; Center for Orthopaedic and Hand Surgery, University Hospital of Strasbourg, Illkirch, France; Novartis Pharmaceuticals AG, Basel, Switzerland; Novartis Healthcare Pvt. Ltd., Raheja Mindspace, Hyderabad, India; Novartis Pharmaceuticals Corporation, East Hanover, NJ USA

## Abstract

Osteomyelitis is a serious infection predominantly caused by Gram-positive bacteria, including methicillin-resistant *Staphylococcus aureus* (MRSA). Orthopaedic device-related infections are complex and require a careful combination of surgical intervention and antimicrobial therapy. Daptomycin, a cyclic lipopeptide, effectively penetrates soft tissue and bone and demonstrates rapid concentration-dependent bactericidal activity against Gram-positive pathogens. This retrospective, non-interventional study evaluated clinical outcomes in patients with osteomyelitis or orthopaedic device infections treated with daptomycin from the European Cubicin® Outcomes Registry and Experience (EU-CORE^SM^) study. Patients were treated between January 2006 and April 2012, with follow-up to 2014. Clinical outcomes were assessed as success (cured or improved), failure or non-evaluable. Of 6,075 patients enrolled, 638 (median age, 63.5 years) had primary infections of osteomyelitis or orthopaedic device infections, 224 had non-prosthetic osteomyelitis, 208 had osteomyelitis related to a permanent or temporary prosthetic device, and 206 had orthopaedic device infections. The most commonly isolated pathogen was *S. aureus* (214 [49.1 %]; 24.8 % were MRSA). Overall, 455 (71.3 %) patients had received previous antibiotic therapy. Patients underwent surgical interventions, including tissue (225 [35.3 %]) and bone (196 [30.7 %]) debridement, as part of their treatment. Clinical success rates were 82.7 % and 81.7 % in *S. aureus* and coagulase-negative staphylococcal infections. Adverse events (AEs) and serious AEs assessed as possibly related to daptomycin were observed in 6.7 % and 1.9 % of patients, respectively. Daptomycin was discontinued by 5.5 % of patients due to AEs and 10 (1.6 %) deaths were reported. In conclusion, daptomycin was effective and safe in patients with osteomyelitis or orthopaedic device infections.

## Introduction

Osteomyelitis is associated with inflammatory changes in bone tissue accompanying bone destruction due to pyogenic organisms. Infections may complicate a variety of factors such as trauma, surgery to the skeleton, and implant replacement surgery [1, 2]. *Staphylococcus aureus* is the most frequently implicated pathogen in osteomyelitis and coagulase-negative staphylococci (CoNS) the most common in prosthetic joint and device-related infections [1, 3]. Other Gram-positive and Gram-negative organisms may be less frequently involved [2]. The most important factor in the development of chronic osteomyelitis is the production of a biofilm [4]. Once established, biofilms are difficult to eradicate due to the lack of drug penetration, drug inactivation, or physiological state of bacteria within the biofilm [5].

Despite advances in medical and surgical therapies, osteomyelitis remains difficult to treat and is responsible for significant morbidity [6]. The risk and severity of infection can be increased by the presence of a foreign body, such as metallic or prosthetic devices [7]. Treatment of osteomyelitis is complex, usually requiring a specific antibiotic regimen and often necessitating surgery for the removal of any infected bone or soft tissue, including temporary or permanent removal of the implant, or a combination of debridement with implant retention and long-term antimicrobial therapy [8, 9]. Using antibiotic combinations and choosing an optimal surgical procedure can eradicate orthopaedic implant-associated infections in 80–90 % of patients [10]. However, relapse rates can be high even after seemingly successful antibiotic treatment [11].

Daptomycin, a cyclic lipopeptide, displays rapid concentration-dependent bactericidal activity against a wide range of Gram-positive pathogens. Daptomycin penetrates bone and synovial fluid effectively [12] and has demonstrated efficacy in animal models of chronic methicillin-resistant *S. aureus* (MRSA) osteomyelitis [13]. Daptomycin is approved in adult patients, at a dose of 4 mg/kg/day for the treatment of complicated skin and soft tissue infections caused by Gram-positive bacteria, and at 6 mg/kg/day for right-sided endocarditis due to *S. aureus* and for bacteraemia associated with complicated skin and soft tissue infections or right-sided endocarditis [14]. The combination of daptomycin and rifampin has been proposed as a potentially good option for treating staphylococcal-biofilm-related infection [15] as these two agents are synergistic in vitro and both disrupt and inhibit biofilm production [16]. This combination could also reduce the likelihood of *S. aureus* resistance, particularly when combined with early surgical debridement [17].

There have been a number of published case series reporting clinical improvement in patients with osteomyelitis treated with daptomycin [18–20]. The Infectious Diseases Society of America (IDSA) guidelines for the management of MRSA infections recognize daptomycin as an antibiotic option for the treatment of osteomyelitis [21] and the IDSA guidelines for the management of prosthetic joint infections also recommend daptomycin as an alternative to oxacillin and vancomycin [22]. Interim data from the first 220 patients with osteomyelitis, non-prosthetic, and temporary or permanent device-related infection, treated with daptomycin and enrolled in the European Cubicin® Outcomes Registry and Experience (EU-CORE^SM^) registry suggested that daptomycin is an effective and well-tolerated treatment option for osteomyelitis with a follow-up of at least 30 days post-treatment [23]. The objective of the present analysis was to evaluate the effectiveness and safety of daptomycin in a lager cohort of patients with primary infections of osteomyelitis, non-prosthetic and prosthetic device-related, or orthopaedic device infections, enrolled in the EU-CORE registry and followed for up to 2 years after the end of daptomycin therapy.

## Methods

### Patients

EU-CORE was a non-interventional, multicentre, retrospective study that collected real-world outcome data from patients receiving at least one dose of daptomycin for the treatment of serious Gram-positive bacterial infections. Among them, all patients with primary infections of osteomyelitis (non-prosthetic and prosthetic device-related) or orthopaedic device infections were included in this analysis.

### Data collection

A protocol and standardised case report form were used to collect demographic and clinical information on patients who had been treated with daptomycin between January 2006 and April 2012. All data were collected from the medical records of the patients enrolled. Patients with osteomyelitis or orthopaedic device infections were followed for up to two years until 2014. The complete data collection methodology has been described previously [24].

The study was conducted according to the ethical principles of the Declaration of Helsinki. The protocol was approved by the health authority and the Institutional Review Board (IRB) or Ethics Committee (EC) in each country and written informed consent was obtained from patients/legally acceptable representatives of patients according to the requirements of the IRB or EC and/or the local data privacy regulations.

### Clinical outcomes

Clinical outcomes were assessed at the end of daptomycin therapy and at the 12- and 24-month follow-up time points according to the following protocol-defined criteria: cured, clinical signs and symptoms resolved, no additional antibiotic therapy was necessary, or infection cleared with a negative culture reported; improved, partial resolution of clinical signs and symptoms and/or additional antibiotic therapy was warranted; failed, inadequate response to daptomycin therapy, worsening or new/recurrent signs and symptoms, need for a change in antibiotic therapy, or a positive culture reported at the end of therapy; and non-evaluable, unable to determine response due to insufficient information. Clinical success was defined as patients who were cured or improved. The time to improvement was also recorded. The reasons for stopping daptomycin therapy and other antibiotics prescribed following daptomycin were also collected [24].

### Safety

The investigators assessed adverse events (AEs) and serious AEs (SAEs) for 30 days post-treatment. All reported AEs, regardless of their relationship to daptomycin, were recorded and their severity was determined by the local investigators.

### Statistical analysis

Statistical analysis was performed using SAS version 9.3 (SAS Institute Inc., Cary, NC, USA). Inferential analyses were not conducted and only descriptive statistics were used. All analyses were considered to be explanatory. Numerical variables were expressed as arithmetic mean, standard deviation, median, minimum, first quartile, third quartile, and maximum for continuous variables. Categorical variables were summarized by absolute and relative frequencies.

## Results

### Patient demographics and clinical characteristics

A total of 6,075 patients were enrolled in the EU-CORE registry, of whom 638 (10.5 %) had osteomyelitis (non-prosthetic and prosthetic device-related) and orthopaedic device infections. The patients were predominantly Caucasian (*n* = 536; 84.0 %) with a median age of 63.5 years (range, 8–93) and a median body weight of 76.0 kg (range, 24–136). Of the 638 patients, 432 (67.7 %) had osteomyelitis and 206 (32.3 %) had orthopaedic device infections. Osteomyelitis was unrelated to prosthesis in 224 (51.9 %) patients, while in 208 (48.1 %) patients, the disease was related to either a permanent (*n* = 160; 37.0 %) or temporary (*n* = 48; 11.1 %) prosthetic device. The most common sites of infection were the knee (*n* = 171; 26.8 %), hip (*n* = 147; 23.0 %), lower extremity (*n* = 94; 14.7 %), foot/ankle (*n* = 83; 13.0 %), and back (*n* = 65; 10.2 %). The most common prosthetic joints were the prosthetic knee (40.4 %) and prosthetic hip (26.6 %). Patient demographics and clinical characteristics including the most common underlying diseases are summarized in Table 1.

**Table 1:** Demographic and clinical characteristics (safety population)

Characteristics	Patients (*N* = 638) [*n* (%)]
Osteomyelitis, non-prosthetic and prosthetic device-related infection	432 (67.7)
Non-prosthetic	224 (51.9)
Permanent prosthetic device-related	160 (37.0)
Temporary prosthetic device-related	48 (11.1)
Orthopaedic device infection [*n* (%)]	206 (32.3)
Age (years)	
Median	63.5
Range	8–93
Gender [*n* (%)]	
Male	372 (58.3)
Race, Caucasian [*n* (%)]	536 (84.0)
Body weight (kg)	
N	622
Median	76.0
Range	24–136
Renal function [*n* (%)]	
Severe renal impairment (CrCl <30 mL/min) at initiation of daptomycin therapy	46 (7.2)
Patients on dialysis at daptomycin initiation	15 (2.4)
Significant underlying diseases (>5 %) [*n* (%)]	
Cardiovascular disease	276 (43.3)
Diabetes mellitus	140 (21.9)
Fractures	101 (15.8)
Pulmonary disease	54 (8.5)
Gastrointestinal disease	52 (8.2)
Renal disease	48 (7.5)
Immunologic/Inflammatory disease	46 (7.2)
Oncologic disease	44 (6.9)
Anatomical site of infection (>5 %) [*n* (%)]	
Knee	171 (26.8)
Hip	147 (23.0)
Lower extremity	94 (14.7)
Foot/ankle	83 (13.0)
Back	65 (10.2)
Any antibiotics used for this infection prior to daptomycin [*n* (%)]	
Yes	455 (71.3)
No	166 (26.0)
Unknown	16 (2.5)
Missing	1 (0.2)

*CrCl* creatinine clearance

### Microbiology

Culture results were available for 534 (83.7 %) patients and were found positive for 436 (81.6 %) of them. The most frequently isolated pathogen was *S. aureus* (*n* = 214; 49.1 %, of which 50.5 % were MRSA), followed by CoNS species (*n* = 153; 35.1 %). The main culture sources of primary pathogens were deep culture tissue (*n* = 199; 31.2 %), blood (*n* = 93; 14.6 %), bone (*n* = 91; 14.3 %), skin swabs (*n* = 52; 8.2 %), needle aspirates (*n* = 40; 6.3 %), and intraoperative tissue biopsies (*n* = 14; 2.2 %). The culture results in the study population are described in Table 2.

**Table 2:** Primary pathogens in patients with positive cultures

Primary pathogens	Patients with positive cultures (*N* = 436) [*n* (%)]
*Staphylococcus aureus*	214 (49.1)
Methicillin-resistant	108 (24.8)
Methicillin-susceptible	88 (20.2)
Methicillin susceptibility unknown	18 (4.1)
Coagulase-negative *Staphylococcus* species	153 (35.1)
*Staphylococcus epidermidis*	104 (23.9)
Other	49 (11.2)
*Streptococcus agalactiae* or group B streptococci	5 (1.1)
*Streptococcus pyogenes* or group A streptococci	3 (0.7)
Viridans streptococci group	3 (0.7)
*Staphylococcus* species - coagulase not specified	4 (0.9)
*Enteroccoccus faecalis*	19 (4.4)
*Enteroccoccus faecium*	7 (1.6)
Vancomycin-resistant (*Enteroccoccus faecalis* or *Enteroccoccus faecium*)	5 (1.1)
*Enteroccoccus* species	3 (0.7)
Other^a^	25 (5.7)

^a^ Includes *Corynebacterium* species, *Streptococcus dysgalactiae, Streptococcus* species, Gram-positive bacilli, Gram-positive cocci, and Gram-negative bacilli

### Prior and concomitant antibiotic therapies

A total of 455 (71.3 %) patients received antibiotic therapy prior to daptomycin treatment. Glycopeptides (*n* = 220; 34.5 %) were most frequently used, followed by fluoroquinolones (*n* = 151; 23.7 %) and penicillins (*n* = 126; 19.7 %). The main reason for a switch to daptomycin therapy was failure (*n* = 190, 29.8 %) of the previous antibiotic. A total of 372 (58.3 %) inpatients and 116 (18.2 %) outpatients received antibiotics concomitantly with daptomycin therapy. Fluoroquinolones and carbapenems were the most common antibiotics used concomitantly with daptomycin.

### Daptomycin prescribing patterns

**Fig. 1:**
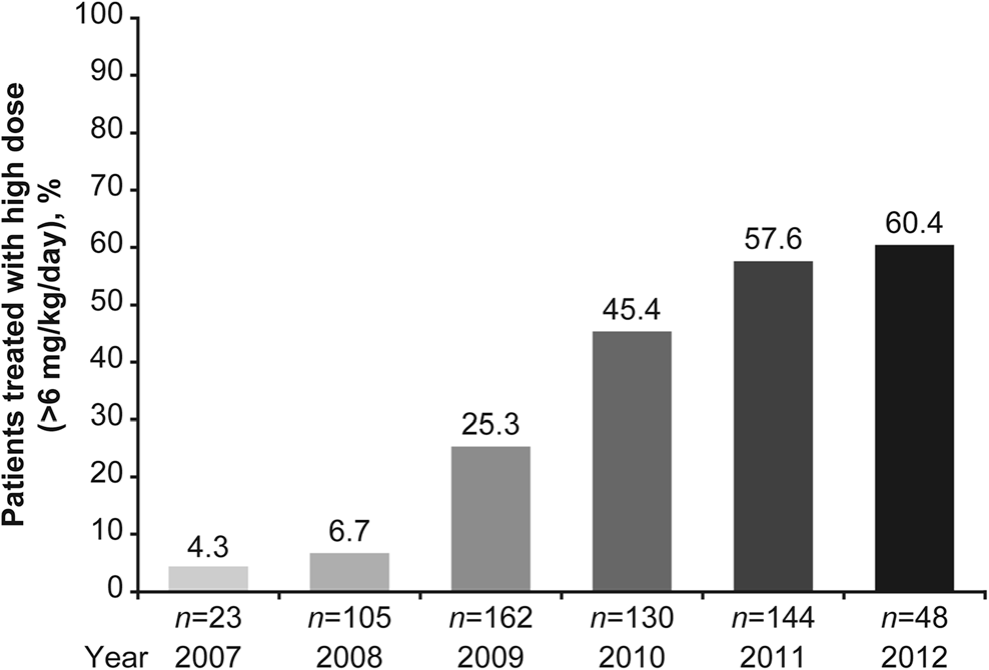
High dose daptomycin use over time in patients with osteomyelitis or foreign body prosthetic infections

Initial doses of 6 mg/kg/day of daptomycin were most frequently prescribed (*n* = 276; 43.3 %), followed by ≥8 to ≤10 mg/kg/day (*n* = 114; 17.9 %), >6 to <8 mg/kg/day (*n* = 92; 14.4 %), and 4 mg/kg/day (*n* = 82; 12.9 %). The other prescribed doses were >4 to <6 mg/kg/day received by 42 (6.6 %) patients and >10 mg/kg/day received by 14 (2.2 %) patients. The initial dosage was unknown for 18 (2.8 %) patients. The median duration of daptomycin therapy was 20 days (range, 1–246). On the basis of patient disposition, the median duration of therapy was 14 days (range, 1–246) for inpatients, 27 days (range, 2–176) for outpatients, and 6 days (range, 1–44) for intensive/critical care patients. The median duration of therapy by type of primary infection was 21 days (range, 1–246) for osteomyelitis and 16 days (range, 1–176) for orthopaedic device infections. A trend towards the use of higher doses over time was noted in the treatment of osteomyelitis and orthopaedic device infections (Fig. 1).

### Surgical interventions

The majority of patients underwent surgery during daptomycin therapy, most commonly tissue/bone debridement (Table 3). Data on the prosthetic device involved and surgical approach were available in a small proportion of patients (Table 4).

**Table 3:** Surgical interventions during daptomycin therapy

Interventions	Patients (*N* = 638) [*n* (%)]^a^
Tissue debridement	225 (35.3)
Bone debridement	196 (30.7)
Foreign device removed	173 (27.1)
Incision and drainage	71 (11.1)
Amputation	23 (3.6)
Other	31 (4.9)
Unknown	1 (0.2)
None	243 (38.1)

^a^ Patients may have had more than one surgical intervention

**Table 4:** Prosthetic device involved and surgical approach

Prosthetic devices / surgical approaches	Patients with prosthetic device involved (*N* = 94) [*n* (%)]
Prosthetic joint	63 (67.0)
Knee	38 (40.4)
Hip	25 (26.6)
Orthopaedic device	29 (30.9)
Permanent	22 (23.4)
Temporary	7 (7.4)
Other	1 (1.1)
Invalid/missing device code	(1.1)
Surgical approach	
Removal without re-implantation	22 (23.4)
Debridement and retention	17 (18.1)
Two-stage exchange	14 (14.9)
One-stage exchange	3 (3.2)
Amputation	2 (2.1)
No surgical approach	36 (38.3)

### Clinical outcomes

**Fig. 2:**
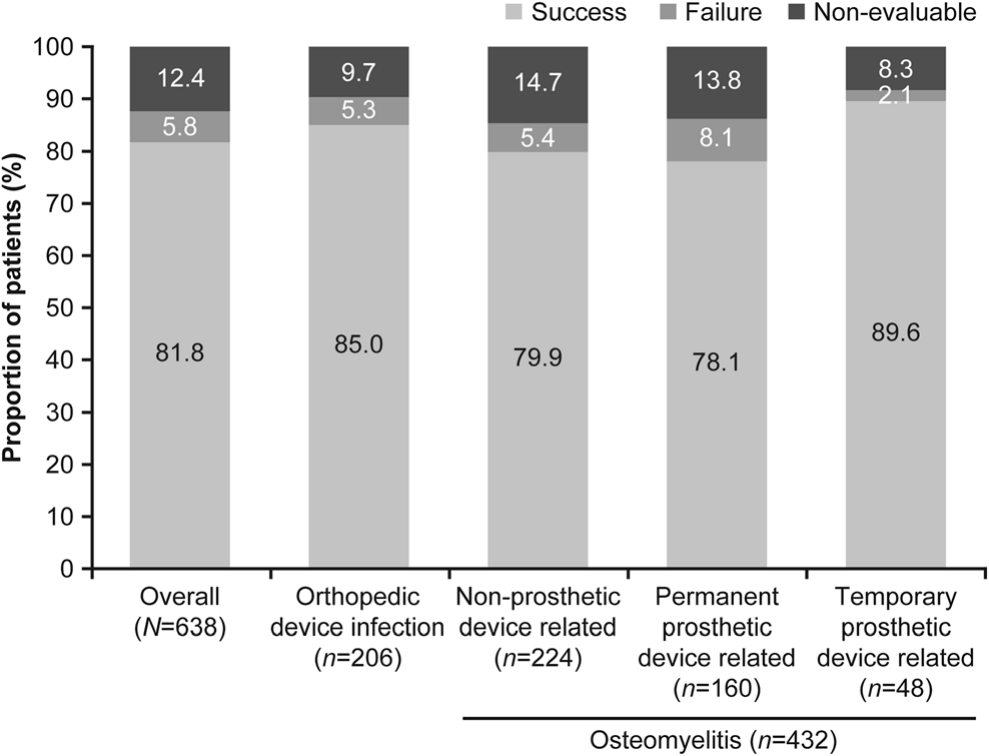
Clinical success rates by primary infection

**Fig. 3:**
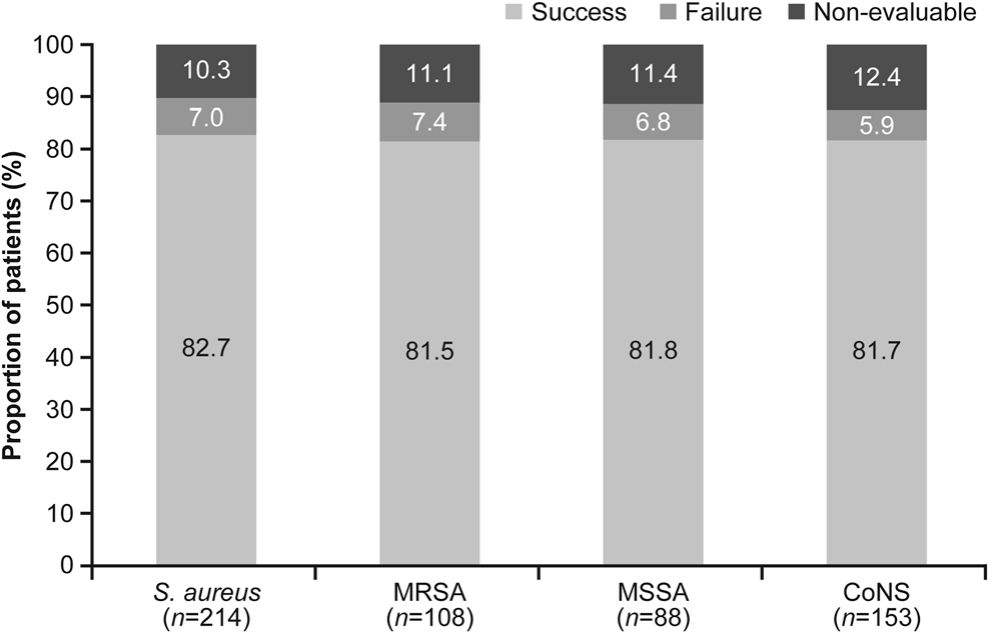
Clinical outcomes by infecting pathogen. *CoNS* coagulase-negative staphylococci, *MRSA* methicillin-resistant *Staphylococcus aureus*, *MSSA* methicillin-susceptible *Staphylococcus aureus*

**Fig. 4:**
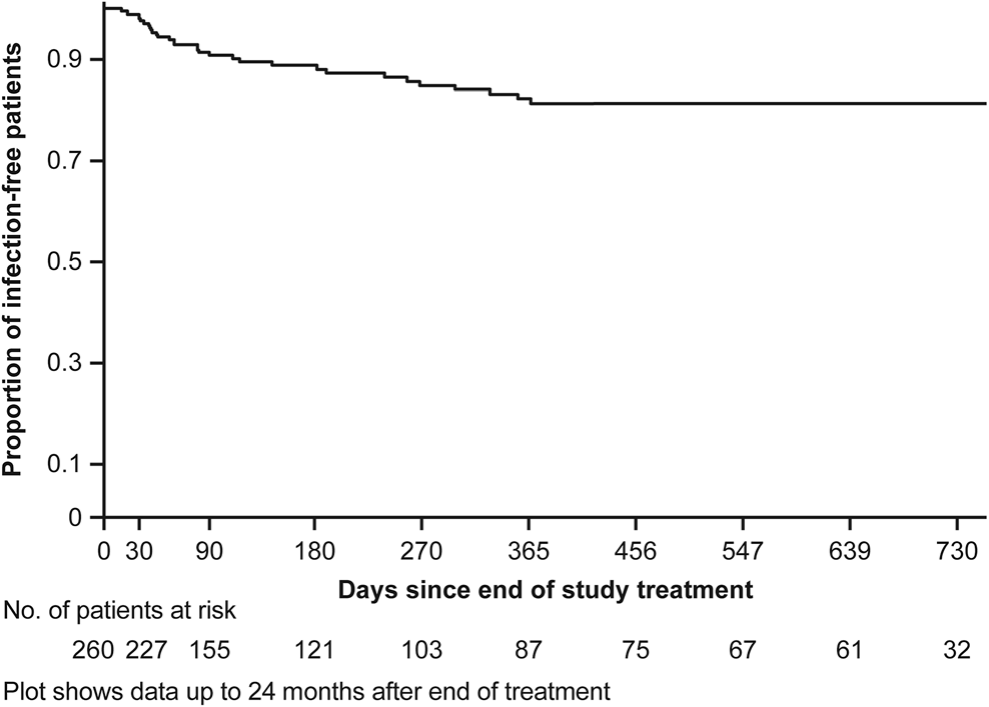
Long-term follow-up of osteomyelitis (non-prosthetic and prosthetic device-related) and orthopaedic device infections; Time to relapse

Overall, clinical success with daptomycin therapy was achieved in 522 (81.8 %) patients. The clinical outcomes by type and subtype of primary infection are summarized in Fig. 2. Clinical success rates were highest in patients with a temporary prosthetic device (89.6 %) as compared with non-prosthetic (79.9 %) and permanent prosthetic device-related osteomyelitis (78.1 %). Clinical success rates were similarly high in patients with *S. aureus* (82.7 %) and CoNS (81.7 %) infections (Fig. 3). Furthermore, high rates of clinical success were observed with both first-line (80.1 %) and second-line daptomycin treatment (83.1 %). Of 318 (49.8 %) patients with data on time to improvement, median time to improvement was 6 days (range, 1–90). Patients receiving daptomycin in combination with rifampin showed numerically higher success rate (*n* = 121; 86.8 %) to those who did not receive rifampin concomitantly (*n* = 401; 80.3 %). Long-term follow-up data were collected from a total of 290 patients. Clinical success in patients followed for up to 2 years was 85.8 %. Most patients (81.3 %) remained relapse-free until the end of the 2-year follow-up period (Fig. 4).

### Safety

Daptomycin-related AEs and SAEs were reported in 43 (6.7 %) and 12 (1.9 %) patients, respectively. Discontinuation of daptomycin due to AEs was reported in 35 (5.5 %) patients and there were ten (1.6 %) deaths (unrelated to study medication) during the study period. The incidence of AEs and SAEs during daptomycin treatment is described in Table 5. Three (0.5 %) patients experienced rhabdomyolysis (two moderate cases and one severe case); all these three events were considered by the investigator as possibly related to daptomycin and two cases were reported as SAEs.

**Table 5:** Adverse events, serious adverse events and deaths during treatment with daptomycin

Safety parameters	Patients (*N* = 638) [*n* (%)]
Any AE(s)	78 (12.2)
AE(s) leading to permanent drug discontinuation	35 (5.5)
AEs related to daptomycin	43 (6.7)
Musculoskeletal and connective tissue disorders	
Rhabdomyolysis	3 (0.5)
Myositis	1 (0.2)
Myalgia	1 (0.2)
AEs occurring in >1 % patients, n (%)	
Blood CPK increased	11 (1.7)
Any SAE(s)	39 (6.1)
SAE related to daptomycin	12 (1.9)
Deaths	10 (1.6)

*AE* adverse event, *CPK* creatine phosphokinase, *SAE* serious adverse event

Serum creatine phosphokinase (CPK) elevation was measured at baseline for 352 (55.2 %) patients and the majority (*n* = 313, 88.9 %) had normal CPK values. Three patients experienced a shift in CPK elevation with a normal value at baseline to >10 × ULN post-baseline. Eleven (1.7 %) patients had elevated CPK levels and were considered by the investigator as AEs related to daptomycin. Of these, 8 (1.3 %) patients discontinued the study drug. The highest CPK levels measured during therapy were reported as normal in most of the patients (*n* = 323, 80.5 %), and above ULN in 78 (24.1 %) patients. Median time for highest CPK was 10 days after starting daptomycin treatment.

## Discussion

In the present analysis, 432 patients with osteomyelitis (non-prosthetic and prosthetic device-related) and 206 patients with orthopaedic device infections were treated with daptomycin. The knee and the hip were the most common sites of prosthetic or orthopaedic device-related infection. Two-thirds of patients with supplementary data underwent surgery as part of their treatment. *S. aureus* was the most frequently isolated pathogen, of which >50 % were methicillin-resistant. The most frequently prescribed dose of daptomycin was 6 mg/kg/day and high-dose daptomycin (>6 mg/kg/day) was used in one-third of the total number of patients.

The study results indicate that overall clinical success rates were high in patients with osteomyelitis (non-prosthetic and prosthetic device-related) and orthopaedic device infections. The majority of patients with non-prosthetic and prosthetic device-related osteomyelitis and orthopaedic device infections, in whom follow-up data were available, remained relapse-free up to 2 years.

Whilst a variety of microbial and host factors are responsible for the development of osteomyelitis, *S. aureus* is the most commonly involved pathogen [6]. Single-agent antimicrobial therapy is generally adequate for the treatment of non-prosthetic device-related osteomyelitis compared to prosthetic device-related osteomyelitis, for which antibacterial combination including rifampicin is commonly used [7]. Infections associated with prosthetic joints cause significant morbidity and account for a substantial proportion of health care expenditures [25]. Despite the use of various oral and parenteral antibiotics against relevant Gram-positive pathogens, treatment remains challenging and relapse rates are high after seemingly successful antibiotic treatment [9, 26]. Osteomyelitis relapses can result from the persistence of a foreign body or incomplete surgical debridement of bone sequestra [7].

Rifampin has excellent oral bioavailability, tissue penetration, and activity in biofilms, and has been extensively used for staphylococcal osteomyelitis in combination with a variety of antimicrobial agents [9]. Daptomycin combined with rifampin is a promising treatment option for implant-associated MRSA infections [15]. To achieve higher success rates, surgical treatment should be combined with a prolonged antibiotic treatment [10, 25]. The optimal dose of daptomycin for treating osteomyelitis and orthopaedic device infections is yet to be defined.

Daptomycin concentrations of 64 μg/ml have demonstrated improved activity against staphylococci embedded in biofilms [27]. High-dose daptomycin (30 mg/kg/day) in guinea pigs, corresponding to 6 mg/kg/day in humans, combined with rifampin (12.5 mg/kg/day) showed superior efficacy against both planktonic and adherent MRSA infections over vancomycin plus rifampin and linezolid plus rifampin combination therapies, and prevented the emergence of rifampin resistance [28].

The clinical efficacy of daptomycin in osteomyelitis is supported by its pharmacokinetic and pharmacodynamic profile. The IDSA guidelines for management of osteomyelitis and prosthetic joint infections recommend daptomycin as an alternative treatment option [21, 22]. High clinical success rates have been reported in patients treated with daptomycin for osteomyelitis in previous studies [18, 19]. Available data support the use of higher doses of daptomycin (8–10 mg/kg/day) in combination with a second drug (usually rifampin) in order to optimize its activity and avoid the emergence of resistance [29].

A favourable safety profile was observed in patients with osteomyelitis and orthopaedic device infections in this analysis. Although serum CPK elevation with daptomycin is well documented, minimal cases were observed in this study and the incidence of muscle related AE was very low. Observational registries have several advantages over other studies, given their inclusive design and ability to demonstrate the real-world clinical experience of treatment. However, they also have limitations such as not being able to control various factors that could influence the treatment outcome. These factors could be the duration of antimicrobial therapy, surgical management strategies, or prior and concomitant antibiotic therapy.

Based on the results of this analysis, daptomycin was found to be effective and safe in patients with osteomyelitis or orthopaedic device infections.
